# Cardiac-Specific Activation of IKK2 Leads to Defects in Heart Development and Embryonic Lethality

**DOI:** 10.1371/journal.pone.0141591

**Published:** 2015-11-05

**Authors:** Bärbel Kraut, Harald J. Maier, Enikö Kókai, Katja Fiedler, Thomas Boettger, Annett Illing, Sawa Kostin, Paul Walther, Thomas Braun, Thomas Wirth

**Affiliations:** 1 Institute of Physiological Chemistry, University of Ulm, Ulm, Germany; 2 Max Planck Institute for Heart and Lung Research, Bad Nauheim, Germany; 3 Institute of Molecular Medicine, University of Ulm, Ulm, Germany; 4 Core Facility Electron Microscopy, University of Ulm, Ulm, Germany; Rutgers University -New Jersey Medical School, UNITED STATES

## Abstract

The transcription factor NF-κB has been associated with a range of pathological conditions of the heart, mainly based on its function as a master regulator of inflammation and pro-survival factor. Here, we addressed the question what effects activation of NF-κB can have during murine heart development. We expressed a constitutively active (CA) mutant of IKK2, the kinase activating canonical NF-κB signaling, specifically in cardiomyocytes under the control of the α-myosin heavy chain promoter. Expression of IKK2-CA resulted in embryonic lethality around E13. Embryos showed defects in compact zone formation and the contractile apparatus, and overall were characterized by widespread inflammation with infiltration of myeloid cells. Gene expression analysis suggested an interferon type I signature, with increased expression of interferon regulatory factors. While apoptosis of cardiomyocytes was only increased at later stages, their proliferation was decreased early on, providing an explanation for the disturbed compact zone formation. Mechanistically, this could be explained by activation of the JAK/STAT axis and increased expression of the cell cycle inhibitor p21. A rescue experiment with an IκBα superrepressor demonstrated that the phenotype was dependent on NF-κB. We conclude that activation of NF-κB is detrimental during normal heart development due to excessive activation of pro-inflammatory pathways.

## Introduction

NF-κB is a pleiotropic transcription factor that has been associated with diverse biological functions such as cell proliferation, cell survival, immunity, and inflammation. Several pathological heart conditions, among them myocarditis, cardiac hypertrophy, adverse cardiac remodeling, and heart failure, have been associated with activation NF-κB signaling as well [1].

NF-κB is a dimeric transcription factor composed of different combinations of the subunits p50, p52, RelA, RelB, and c-Rel. Under baseline conditions, these NF-κB dimers remain inactive and sequestered in the cytoplasm by inhibitory κB (IκB) proteins. Different signals, such as engagement of cytokine or Toll-like receptors, trigger a signaling cascade that eventually converges on and activates the IκB kinase complex (IKK). This complex is composed of the catalytic subunits IKK1 (also known as IKKα) and IKK2 (IKKβ), and the regulatory subunit NEMO (IKKγ). IKK in turn can phosphorylate IκB proteins and thereby mark them for ubiquitin-mediated degradation. This releases NF-κB dimers, which are now free to translocate to the nucleus and activate specific target genes [2].

Murine heart development begins with the specification of cardiac progenitor cells at E6.0 and the formation of the cardiac crescent at E7.5 [3]. Beginning at E8.0, a linear heart tube is formed, sarcomeres are established, and a heart beat can be detected. After cardiac looping with the establishment of left-right asymmetry around E9.0, the heart tube differentiates into the four chambers and undergoes trabeculation and expansion (E10.5—E12.5), followed by further maturation of essential components such as the heart valves and the great vessels (E13.5 onwards).

Genetic and genomic approaches have identified a multitude of genes essential for mammalian heart development. In particular, they have revealed a complex network of cardiogenic transcription factors that orchestrate heart development [3]. Components of the IKK/ NF-κB signaling pathway appear not to be essential for heart development: For example, mice deficient in IKK2 (*Ikbkb* ^*-/-*^) undergo normal heart development and only die due to hepatocyte apoptosis between E12.5 and E14.5 [4–6]. Similarly, a conditional ablation of IKK2 in the heart—using the myosin light chain 2V promoter which is specifically active in cardiomyocytes—does not affect normal heart development [7], neither does direct interference with the NF-κB pathway by expressing a transgenic IκBα superrepressor [8].

Still, IKK/ NF-κB signaling may be of great importance under pathologic conditions, since IKK/ NF-κB is activated during many adverse events that are common during gestation, such as infection. In this study, we wanted to address the consequences of the activation of IKK/ NF-κB in the developing heart as a model for adverse events during gestation.

## Materials and Methods

### Mice

Mice were kept under specific pathogen-free conditions at the animal facility of the University of Ulm. The tetO.IKK2-CA [9], α-MyHC.tTA [10], and IκBα-3M mice [11] have been described previously. Animals were killed by cervical dislocation under isoflurane anesthesia. All experiments were in accordance with institutional guidelines and German animal protection laws and were approved by the regional government authority (Regierungspräsidium Tübingen).

### Histology and immunofluorescence microscopy

For paraffin sections, whole embryos were fixed in buffered formalin, dehydrated, embedded in paraffin, and cut in 3 μm sections on a rotary microtome (Thermo Scientific). Sections were deparaffinized and rehydrated, and then used for H&E staining. Where appropriate, sections were boiled in citrate buffer for 10 minutes prior to immune staining. For cryosections, embryos snap-frozen in liquid nitrogen were cut in 4 μm sections on a cryotome (Leica). Sections were warmed and immediately fixed with PBS/ 4% paraformaldehyde for 10 min. Prior to antibody staining, both cryosections and paraffin sections were blocked with 5% BSA in TBS for 1 hour at room temperature. First antibody incubation was done at a dilution of 1:100–1:200 (Dako antibody diluent) for 1 h at room temperature, with antibodies against α-SMA (Sigma F3777), IKK1/2 (Santa Cruz sc-7607, lot H2208), cardiac Troponin T (Abcam 8295), RelA (NeoMarkers RB-1638-P1), sarcomeric actinin (Sigma A7811), desmin (Cell Signaling 4024), F4/80 (eBioscience 14-4801-85, lot E04273-301), Icam-1 (Santa Cruz sc-18853, lot A2808), Sca-1 (eBioscience 14-5981-82, lot E020936). Secondary antibodies coupled with Alexa Fluor 488 or 596 were purchased from Invitrogen/ Molecular Probes, and were incubated for one hour at room temperature. Co-staining was performed with 4',6-diamidino-2-phenylindole (DAPI) for nuclear staining. For detection of apoptosis, an in situ TUNEL detection kit (Roche) was used. Fluorescent samples were analyzed on a Zeiss Axiovert 200M microscope equipped with a digital camera (Zeiss AxioCam MR3) and Axiovision software. Other staining were evaluated on a Leica DM5500B microscope equipped with a DFC420C camera (Leica).

### Murine cardiomyocyte isolation

Excised hearts were digested in a calcium-free buffer supplemented with 6.5 mg/mL Liberase DH (Roche) and 3.5 mg/mL trypsin (Sigma). Digestion was stopped with FCS, and the cell suspension was filtered through a 100 μm mesh and centrifuged.

### BrdU labeling and flow cytometry

Pregnant females were given BrdU (100 mg/ kg body weight i.p.) 4 hours before harvesting the embryos. FACS of digested whole hearts was performed on a FACSCanto™ II equipped with FACSDiva 6.2 software (BD).

### Protein extracts and Western blotting

Tissue was snap-frozen in liquid nitrogen, pulverized, and resuspended in a buffer containing 4% SDS, 100 mM Tris-HCl, and protease/ phosphatase inhibitors (Roche). 10 to 20 μg of protein were separated on 4–12% gradient gels (Invitrogen). Proteins were transferred to a PVDF or nitrocellulose membrane with a semi-dry blotter (Bio-Rad). Membranes were blocked for 1 h (RT) in TBS, 0.1% Tween-20 with 5% w/v nonfat dry milk, incubated with primary antibody over night (6°C) in TBS, 0.1% Tween-20 with 5% nonfat dry milk or 5% BSA, and then incubated with HRP-coupled secondary antibody for 1 h (RT). After application chemiluminescence reagent, membranes were exposed to x-ray films. Primary antibodies against the following antigens were used: human IKK2 (Abcam Y466), IKK1/2 (Santa Cruz sc-7607, lot H2208), ERK2 (Santa Cruz sc-154, lot F0210), p21 (Santa Cruz sc-6246, lot A1209), Stat1 (Cell Signaling 9172, lot 14), phospho-Stat1 (Cell Signaling 9167, lot 6), and luciferase (Promega G7451).

### RNA extraction and quantitative PCR

Heart tissue was snap-frozen in liquid nitrogen, pulverized and processed with the Qiagen RNeasy Fibrous Tissue kit. The Transcriptor High Fidelity cDNA Synthesis Kit (Roche) was used for cDNA synthesis. Quantitative PCR was done on a LightCycler 480 system, using the Universal Probe Library (Roche). *Rpl13* was used as reference gene for relative quantification. Primer sequences are available on request.

### Gene expression profiling

RNA quality was checked on the Agilent 2100 Bioanalyzer. 200 ng total RNA was amplified and labeled with the Whole Transcript Sense Target Labeling Assay (Affymetrix) using the GeneChip protocol. Labeled samples were hybridized to Affymetrix GeneChip^®^ Mouse Gene 1.0 ST Array and further processed. Arrays were scanned with an Affymetrix GeneChip Scanner 3000 7G and data were analyzed by the RMA algorithm using the Affymetrix Expression Console and the GeneSifter^®^ microarray data analysis system. Microarray data are available in the ArrayExpress database (www.ebi.ac.uk/arrayexpress) under accession number E-MTAB-3971 (http://www.ebi.ac.uk/arrayexpress/experiments/E-MTAB-3971).

### Statistical analysis

Values are given as arithmetic mean + (or +/-) standard error of the mean (SEM). Means of two groups were compared by the student’s t test or, when indicated, by the Mann-Whitney test. Welch correction was applied for unequal variances. Means of multiple groups were compared by ANOVA. All tests were two-tailed. p values <0.05 were deemed significant (*p<0.05, **p<0.01, ***p<0.001).

## Results

### Expression of Constitutively Active IKK2 in the Heart Causes Embryonic Lethality

**Fig 1:**
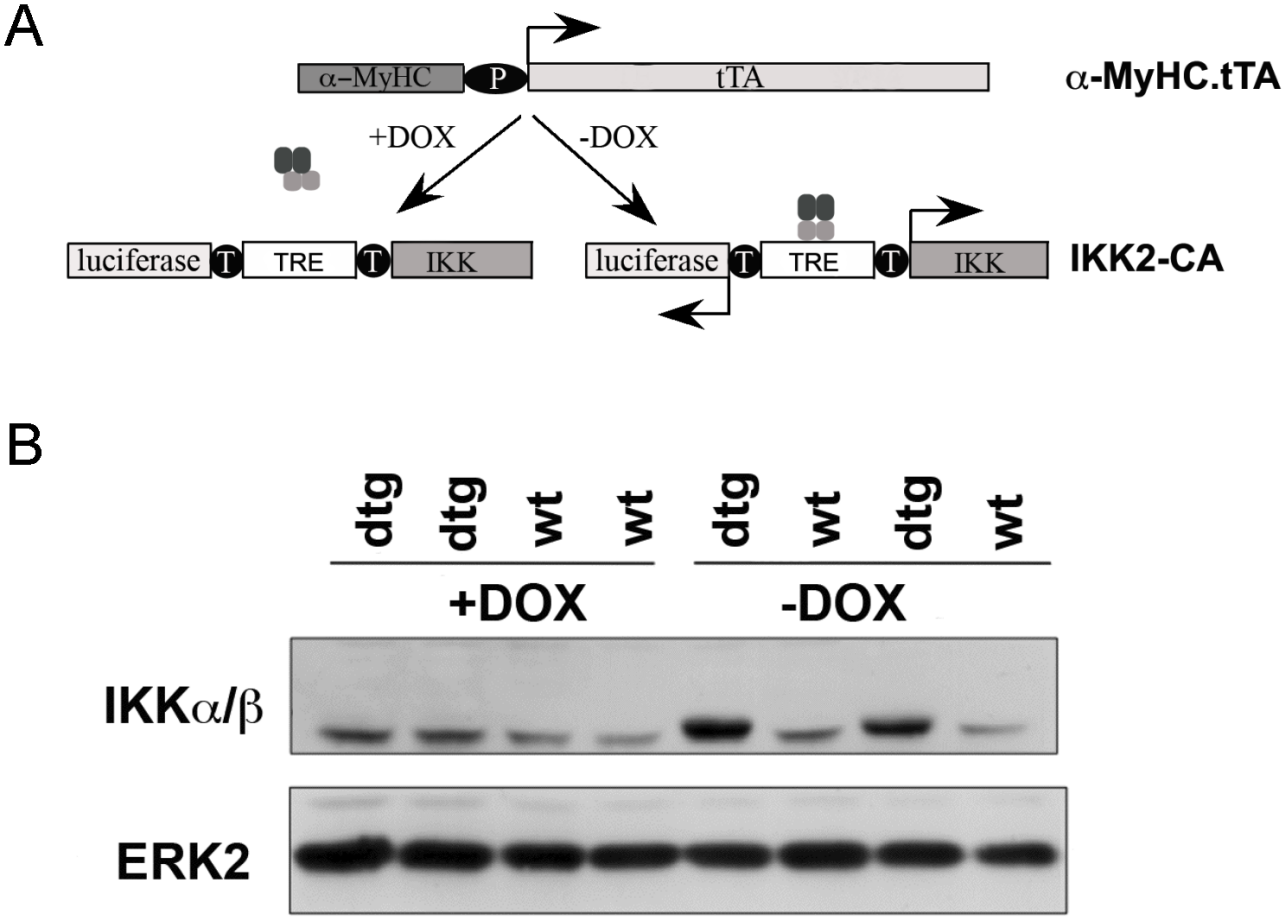
Cardiomyocyte-specific expression of IKK2-CA in embryos. (A) In order to direct the expression of constitutively active IKK2 (IKK2-CA) to the heart, mice expressing the tetracycline-transactivator (tTA) under the α-myosin heavy chain promoter (α-MyHC) were crossed with mice bearing an IKK2-CA (IKK) and a luciferase allele under the control of a bidirectional tTA-responsive promoter (TRE, tetracycline response element). The transgene IKK2-CA is expressed only in the absence of doxycycline (-DOX), whereas its expression is blocked in the presence of doxycycline (+DOX). (B) Western blot of embryonic heart extracts at day E12.5. The transgene IKK2-CA is expressed exclusively in double transgenic (dtg) IKK^MyHC^ animals in the absence of doxycycline (-DOX). The antibody used also detects endogenous IKK, which migrates slightly below the transgene and is readily detectable in wild type animals (wt) as well as IKK^MyHC^ animals (dtg) treated with doxycycline. An antibody against ERK2 was used as a loading control.

In order to activate NF-κB in the developing myocardium, constitutively active IKK2 (IKK2-CA) was expressed specifically in cardiomyocytes. To this end, mice expressing the tetracycline transactivator (tTA) under the control of the cardiomyocyte-specific α-myosin heavy chain (α-MyHC) promoter [10] were crossed with mice carrying a constitutively active IKK2 transgene (IKK2-CA) and a luciferase reporter gene regulated by a bidirectional, tTA-responsive promoter (Fig 1A) [9]. As expected, expression of the IKK2 transgene was only seen in hearts of double transgenic (IKK^MyHC^) embryos and could be silenced by the administration of doxycycline to pregnant mothers (Fig 1B).

When mice were bred in the absence of doxycycline, i.e. under conditions that allow the transgene to be expressed, the number of double transgenic animals was strongly reduced in comparison to single transgenic and non-transgenic animals: Only 3.3% (instead of the expected 25%) of the born animals (4 out of 122) carried both transgenes (Table 1). When the transgene expression was shut off during development by administering doxycycline to the pregnant females, the ratio was normalized to the expected 25% [12]. These observations suggested that the expression of constitutively active IKK2 resulted in embryonic lethality.

**Table 1:** Expression of constitutively active IKK2 in cardiomyocytes results in embryonic lethality.

MyHC-tTA	IKK2-CA	Numbers observed	Numbers expected	% observed	% expected
-	-	40	30.5	32.8	25
+	-	40	30.5	32.8	25
-	+	38	30.5	31.1	25
+	+	4	30.5	3.4	25
**Total**		122	122	100	100

The table shows the absolute number and the percentage of animals born with the indicated genotypes for the transgenes MyHC.tTA and IKK2-CA (observed), and the corresponding expected number and percentage (expected). The observed number of animals positive for both MyHC-tTA and IKK2-CA (4) is much lower than expected (30.5).

**Fig 2:**
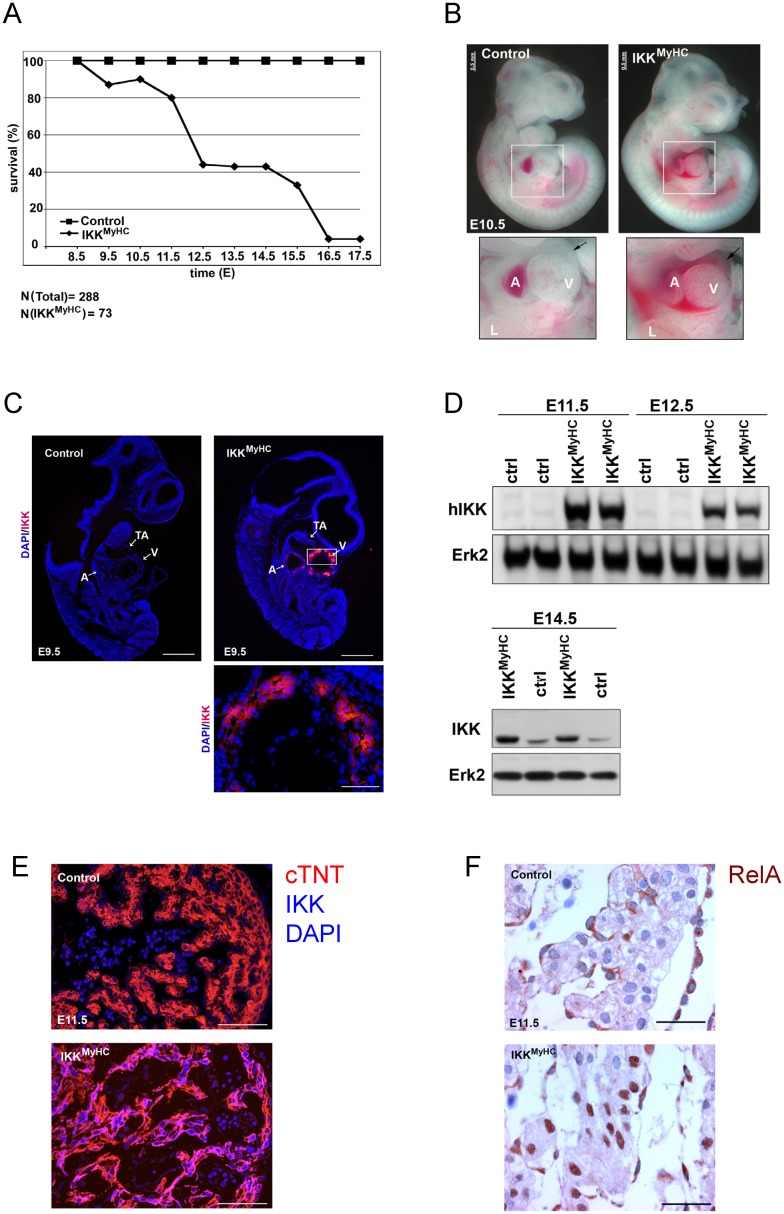
Cardiomyocyte-specific expression of IKK2-CA is embryonically lethal. (A) Survival rate of IKK^MyHC^ embryos with expression of IKK2-CA transgene (i.e. in the absence of doxycycline). Pregnant mothers were killed at the indicated day of embryonic development, embryos were checked for viability by assessing the presence of a heart beat, and the genotype of the embryos was assessed both by PCR and by measuring luciferase activity. A total of 73 IKK^MyHC^ embryos were scored out of 288 embryos in total at the indicated days of embryonic development. Controls comprise both single transgenic and non-transgenic embryos. (B) Gross appearance of embryos at E10.5. Note the pericardial edema in IKK^MyHC^ embryos (arrow, lower right); the pericardium is filled with blood (lower right). A: atrium, V: ventricle, L: limb. Scale bar: 0.5 mm. (C) Immunofluorescence analysis of IKK2-CA transgene expression in control (upper left) and IKK^MyHC^ (upper right) embryos at E9.5. IKK2-CA expression (red) was exclusively detected in the heart of IKK^MyHC^ embryos. Nuclear staining with DAPI (blue). Scale bar 500 μm. Lower panel: Magnification of the ventricle of an IKK^MyHC^ embryo. Scale bar: 100 μm. A: atrium, V: ventricle, TA: truncus arteriosus. (D) Upper panel: Western blot analysis of heart extracts from E11.5 and E12.5 embryos. An antibody against human IKK (hIKK) was used to detect the IKK transgene, which is of human origin. IKK2-CA was exclusively detected in double transgenic embryos (IKK^MyHC^), but not in control embryos (ctrl). Expression of Erk2 was used as a loading control. Lower panel: Western blot analysis of heart extracts of E14.5 animals with an antibody detecting both human (transgenic) and murine (endogenous) IKK. Erk2 was used as a loading control. (E) Immunofluorescence staining of E11.5 cryosections with antibodies against cardiac troponin T (cTNT, red), IKK and DAPI (blue), showing a colocalization of the cardiomyocyte-specific cTNT and the IKK transgene in IKK^MyHC^ embryos. Scale bar 200 μm. (F) Immunohistochemical analysis at E11.5 shows that RelA/p65 in IKK^MyHC^ cardiomyocytes, but not in control cardiomyocytes, is localized in the nucleus, suggesting NF-κB activation. Scale bar: 310 μm.

To determine the time point of lethality, the viability of embryos was analyzed at different time points of gestation. IKK^MyHC^ embryos started to die as early as E9.5 (Fig 2A). The exact time point of death was somewhat variable between individual embryos, but 50% of the animals were dead between E12.5 and E13.5. Less than 4% of IKK^MyHC^ embryos survived longer than E17.5. Closer examination of the heart showed pericardial edema and hemorrhages at the ventricles by E10.5; in several cases the pericardial sac was filled with blood (Fig 2B).

Since the lethality was observed as early as day E9.5, we confirmed by immunofluorescence that the transgene IKK2-CA was actually expressed at this early stage (Fig 2C). Transgene expression was also detectable by Western blot at later time points and was stronger than endogenous IKK2 expression (Fig 2D). Expression was restricted to cardiomyocytes, as shown by immunofluorescence costaining of sections at E11.5 with an antibody against the human IKK2 transgene and an antibody against the cardiomyocyte-specific protein cardiac troponin T (Fig 2E). Next, we wanted to analyze whether IKK2-CA expression actually activated NF-κB signaling. Indeed, nuclear translocation of RelA, a marker for classical NF-κB activation, was found in cardiomyocytes of IKK^MyHC^ embryos, whereas this was not the case for controls (Fig 2F).

### IKK^MyHC^ embryonic hearts show defects in compact zone formation

**Fig 3:**
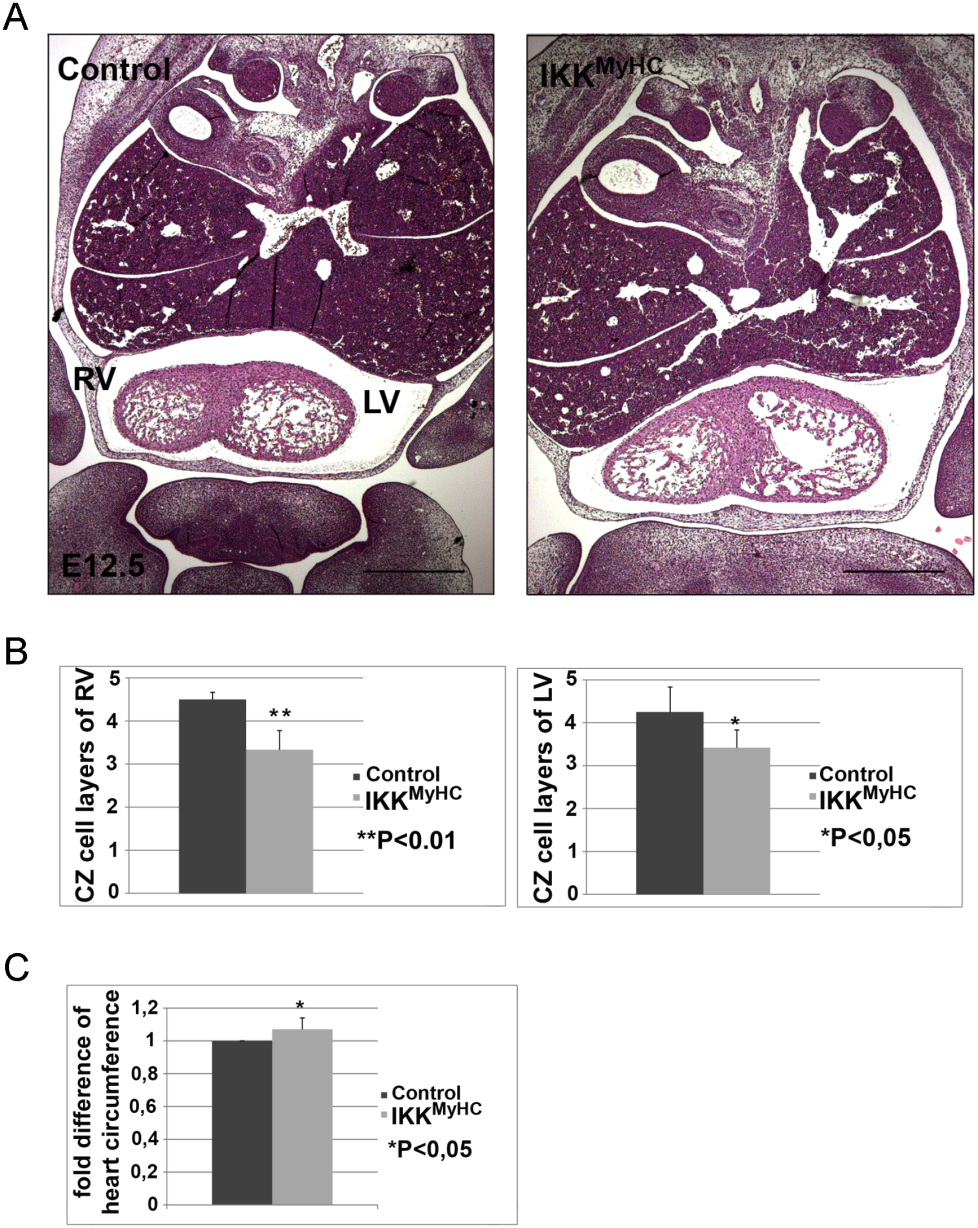
Cardiomyocyte-specific expression of IKK2-CA leads to defects in compact zone formation. (A) Hematoxylin/ eosin staining of frontal paraffin sections of control (left) and IKK^MyHC^ embryos (right) at E12.5. RV, right ventricle; LV, left ventricle. Scale bar: 500 μm. Both ventricles of the IKK^MyHC^ embryo show fewer cell layers in the compact zone, and the IKK^MyHC^ heart is generally dilated. (B) Quantification of the number of compact zone cell layers of the right and left ventricles of control and IKK^MyHC^ hearts. N = 6 embryos per group; shown are the arithmetic means +SEM. *P<0.05, **P<0.01. (C) Quantification of total heart circumference of control and IKK^MyHC^ hearts. N = 6 embryos per group; shown are the arithmetic means +SEM. *P<0.05.

Embryonic hearts are characterized by the presence of an inner trabecular and an outer compact zone. Systematic histological examination of the IKK^MyHC^ embryonic hearts at E12.5 showed a significant reduction in the thickness of the compact zone: The compact zones of both ventricles comprised fewer cell layers in comparison to wild type animals (Fig 3A and 3B). Furthermore, measurement of ventricle circumference revealed that the hearts of IKK^MyHC^ animals were slightly enlarged (Fig 3C).

### IKK^MyHC^ embryonic hearts show defects in the contractile apparatus

**Fig 4:**
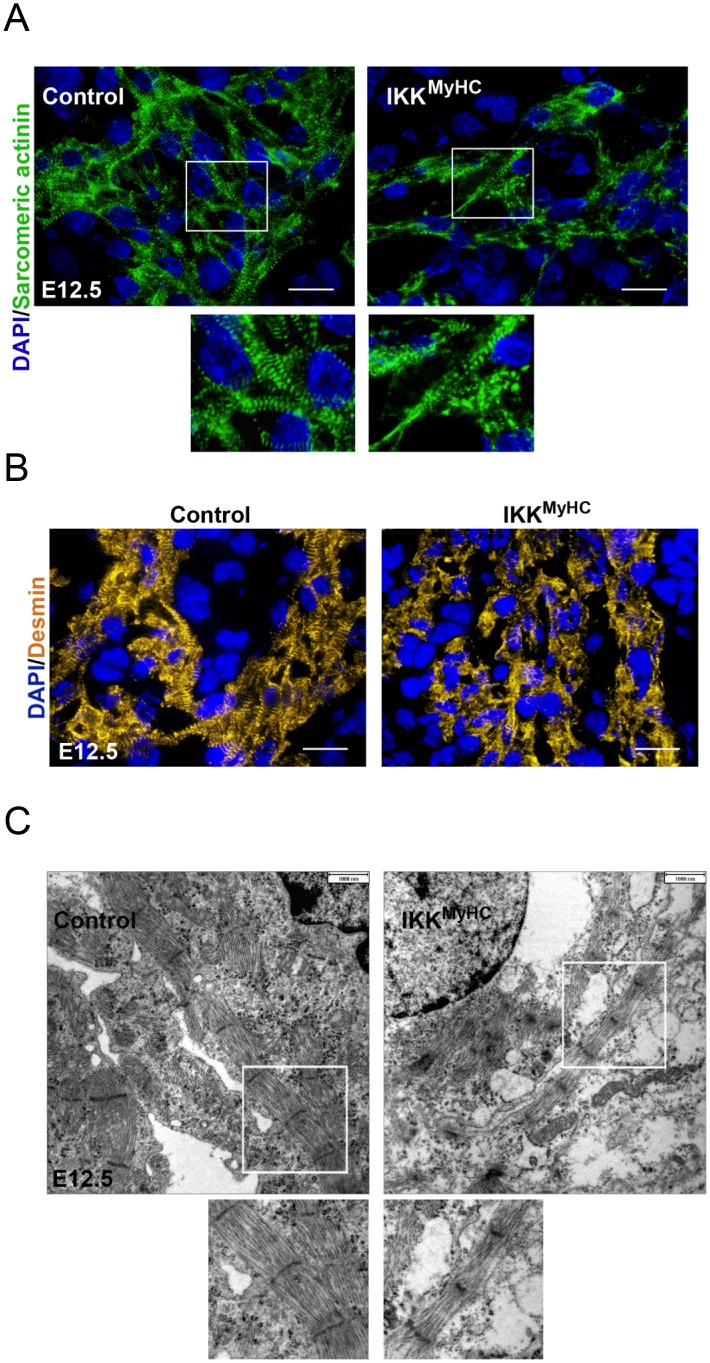
Cardiomyocyte-specific IKK/NF-κB activation induces myofiber degeneration and loss of typical cardiomyocyte proteins. (A) Control and IKK^MyHC^ sections were stained for sarcomeric α-actinin (green) and DAPI (blue). Scale bar: 100 μm. (B) Control and IKK^MyHC^ sections were stained for desmin (yellow) and DAPI (blue). Scale bar: 100 μm. (C) Electron microscopic analyses of control and IKK^MyHC^ heart sections. Cardiomyocytes of control animals show normally developed myofibers with typical sarcomeres (left). IKK^MyHC^ cardiomyocytes show general disorganization and a reduced width of myofibers (right). Scale bar: 1 μm.

The enlargement of embryonic IKK^MyHC^ hearts prompted us to examine the contractile apparatus more closely. As evident from sarcomeric α-actinin staining, hearts of IKK^MyHC^ embryos at E12.5 exhibited a defective organization of the contractile filaments (Fig 4A). Whereas control hearts showed the characteristic striped pattern of sarcomeric α-actinin, the staining of IKK^MyHC^ hearts was much more diffuse, with only few cardiomyocytes retaining the regular striped pattern. Similarly, the staining for the Z disc protein desmin was more diffuse and scattered in IKK^MyHC^ hearts as compared to control hearts (Fig 4B). Ultrastructural analysis by transmission electron microscopy confirmed the morphological abnormalities of IKK^MyHC^ cardiomyocytes (Fig 4C). Control embryos showed the typical pattern of myofibers with normally developed sarcomeres. In contrast, IKK^MyHC^ animals exhibited generally thinner and more disorganized myofibers.

### Embryonic IKK^MyHC^ hearts exhibit inflammation

**Fig 5:**
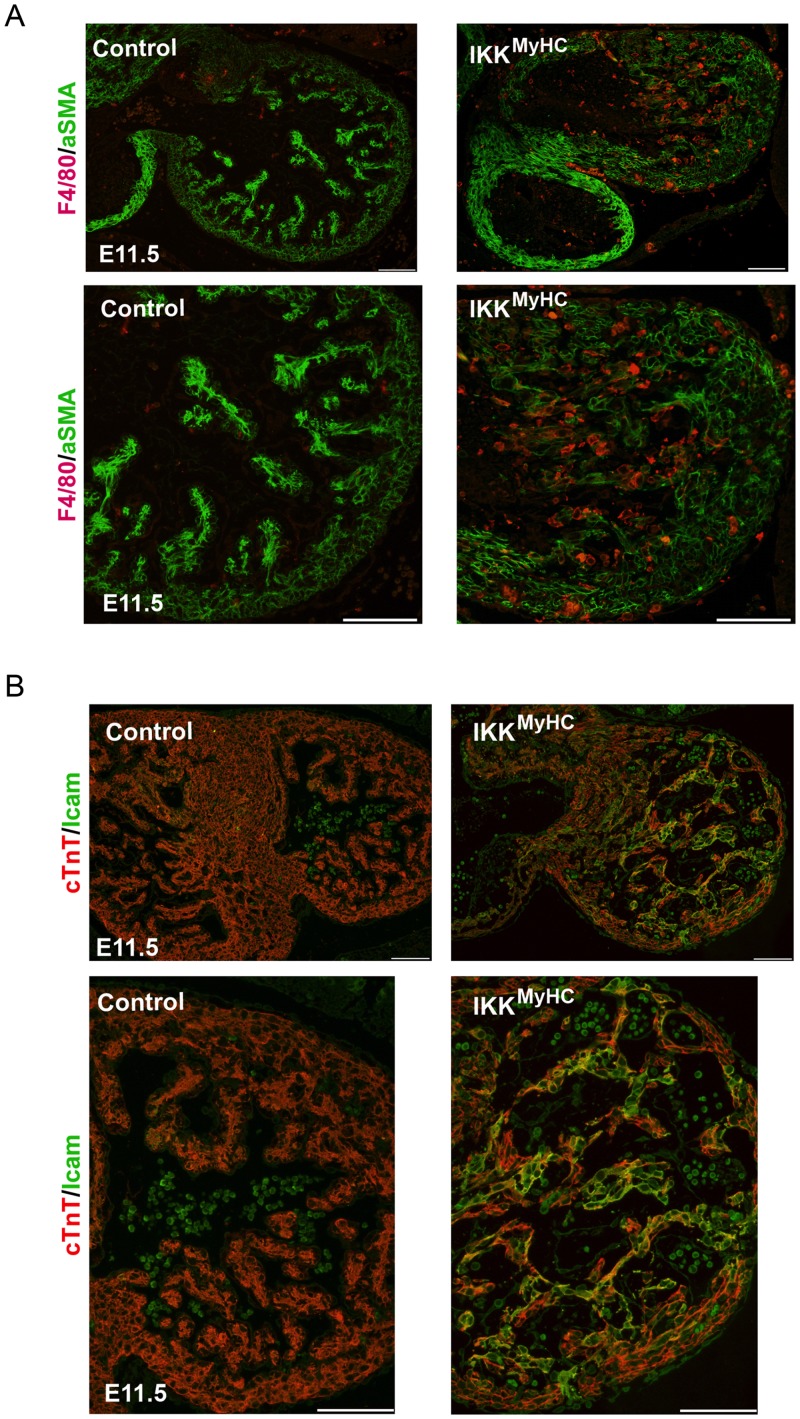
Cardiomyocyte-specific IKK/NF-κB activation up-regulates cell-adhesion molecules and induces the recruitment of macrophages. (A) Immunofluorescence staining of heart cryosections from control and IKK^MyHC^ embryonic hearts shows infiltration with F4/80 positive macrophages. Macrophages were stained with an antibody against F4/80 (red) and cardiomyocytes were stained with an antibody against α-smooth muscle actin (αSMA, green). Scale bar: 100 μm. (B) Immunofluorescence staining of heart cryosections from control and IKK^MyHC^ embryonic hearts show Icam-1 expression in IKK^MyHC^ animals, but not in control animals at E11.5. Colocalization of Icam-1 (green) and the cardiomyocyte-specific cTNT (red) is visible as yellow staining. Scale bar: 100 μm.

Since the IKK/ NF-κB axis is one of the master regulators of inflammation in adults, embryonic IKK^MyHC^ hearts were analyzed for features of inflammation. Indeed, IKK^MyHC^ hearts at E11.5 manifested a strong infiltration of macrophages, as visualized by staining for the macrophage marker F4/80 (Fig 5A). Also, adhesion molecules like ICAM-1 were strongly expressed in cardiomyocytes (Fig 5B). This indicated that IKK^MyHC^ hearts were characterized by continuous recruitment of inflammatory cells.

### Cardiomyocyte apoptosis is enhanced in embryonic IKK^MyHC^ hearts at later stages

**Fig 6:**
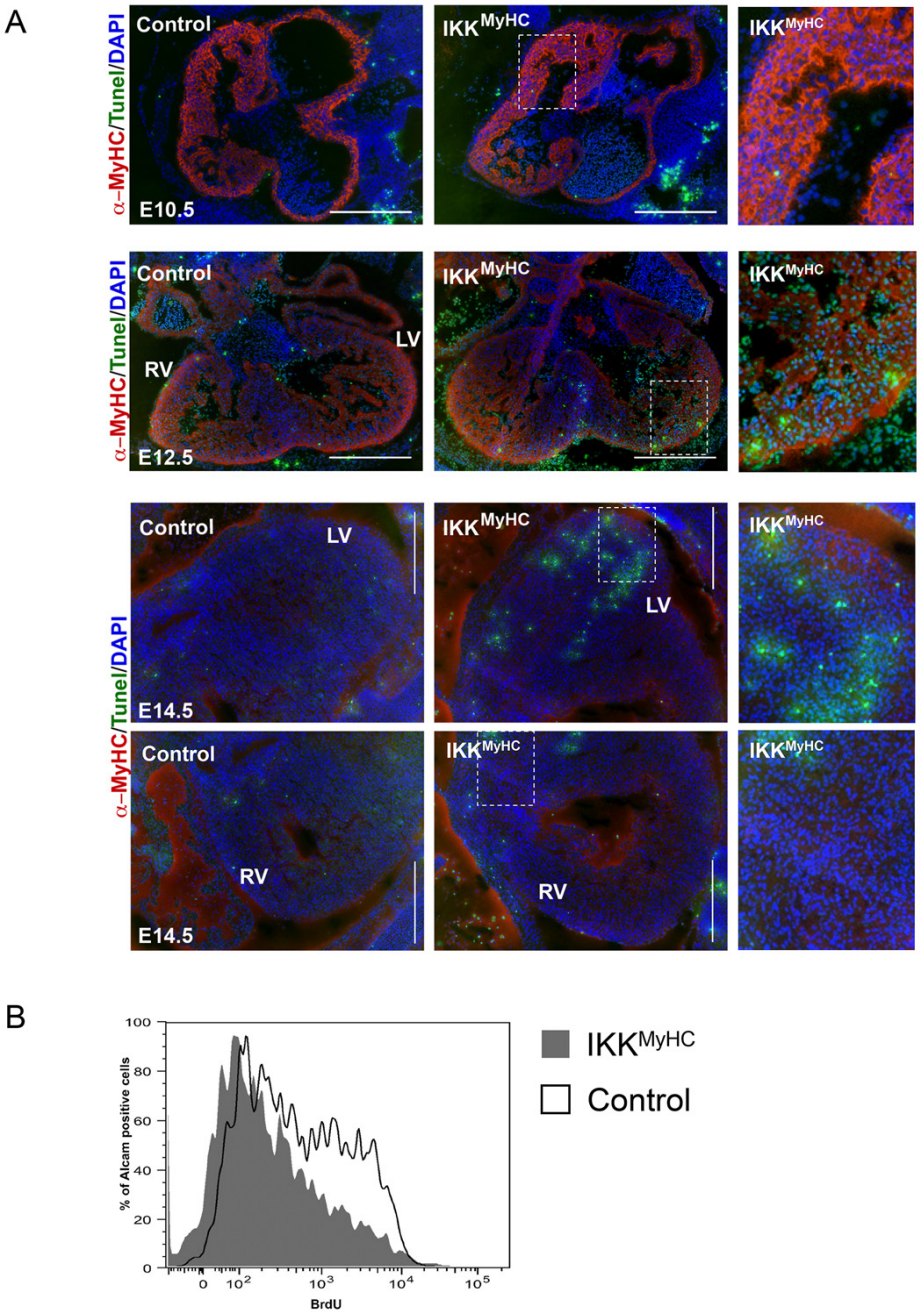
Cardiomyocyte undergo apoptosis only in later stages of IKK2-CA expression, but show early defects in proliferation. (A) TUNEL (terminal deoxynucleotidyltransferase-mediated UTP end labeling) assays were performed at E10.5, E12.5 and E14.5 for the indicated genotypes. TUNEL-positive cells show green fluorescence. An antibody against α-myosin heavy chain was used to specifically stain cardiomyocytes (red); nuclei were stained with DAPI (blue). RV, right ventricle; LV, left ventricle. Scale bar: 500 μm. The right panels show higher magnifications of the areas indicated in the middle panels. (B) Pregnant mice were injected with BrdU and their embryos (E10.5) were harvested 4 hours later. Cardiomyocytes were isolated and analyzed in FACS by staining with antibodies against the early murine cardiomyocyte marker Alcam (CD166)[13] and against BrdU. IKK^MyHC^ cardiomyocytes (grey, filled) generally show a shift to the left in comparison to control cardiomyocytes (black line), indicating a lower BrdU staining and thus less proliferation.

The reduced thickness of the compact zone of embryonic IKK^MyHC^ hearts could be the result of enhanced cell death and/ or decreased proliferation. Embryonic hearts were examined for apoptosis at days E10.5, E12.5 and E14.5 using TUNEL (terminal deoxynucleotidyltransferase-mediated UTP end labeling) assays (Fig 6A). Apoptosis was prominent in IKK^MyHC^ embryos only at later time points, thus putting into question its causal role for the reduced thickness of the compact zone. In contrast, proliferation appeared to be reduced already at day E11.5, as revealed by BrdU labeling of cardiomyocytes (Fig 6B).

### Gene Expression Analysis of embryonic IKK^MyHC^ hearts

**Fig 7:**
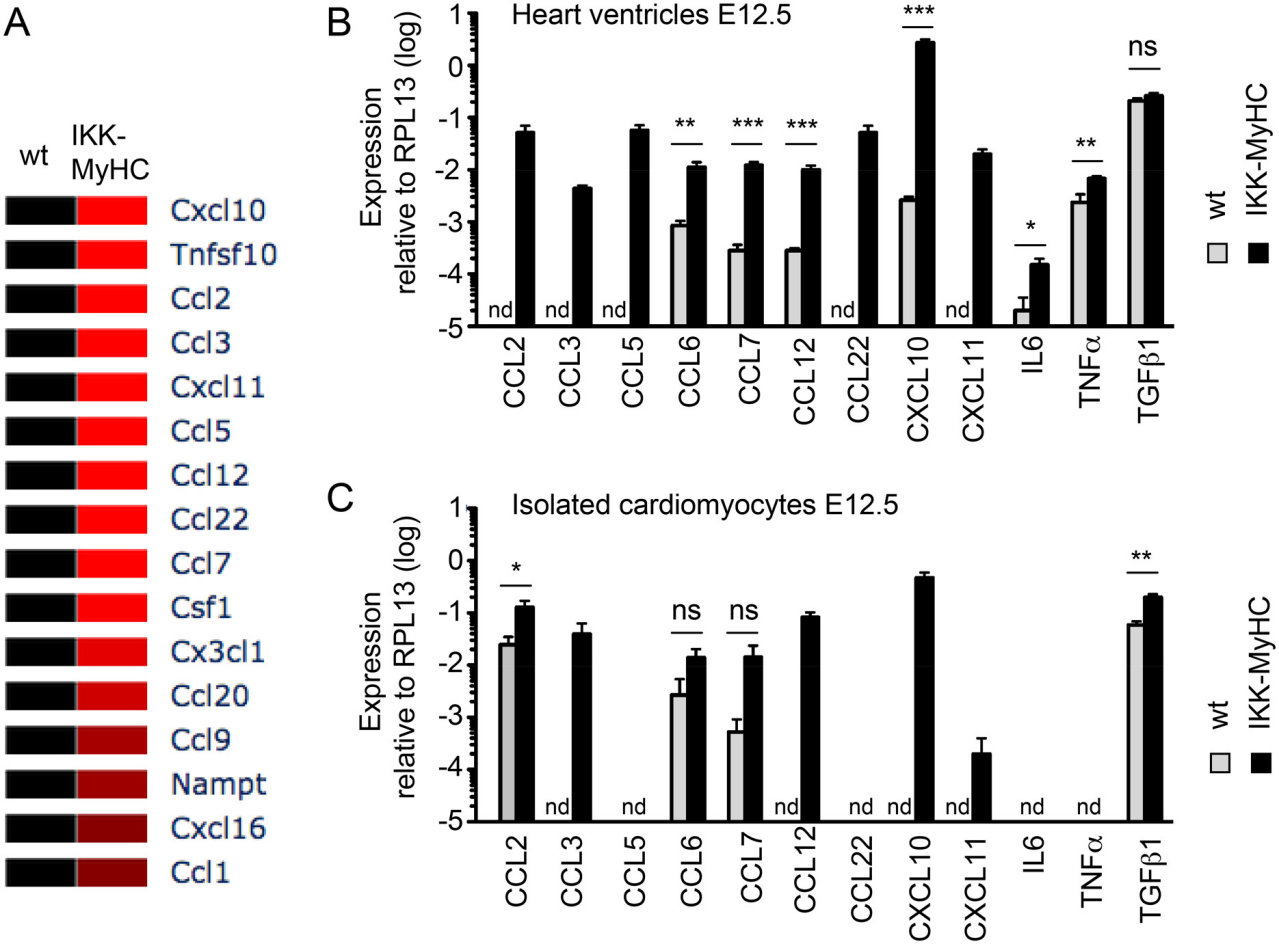
Cytokine profile of IKK^MyHC^ hearts and IKK^MyHC^ cardiomyocytes. (A) Heat map of cytokines expressed in IKK^MyHC^ hearts at E12.5 (>2-fold regulation, p<0.05) as detected by microarray (N = 3 for wild type (wt), N = 4 for transgenics (IKK-MyHC). (B) Expression of the indicated cytokines as assessed by real-time quantitative PCR from IKK^MyHC^ (black bars) and control (grey bars) heart ventricles at E12.5. Shown are the means +SEM; N = 6, *P<0.05, **P<0.01, ***P<0.001 (t test), nd, not detected. (C) Expression of the indicated cytokines as assessed by real-time quantitative PCR from IKK^MyHC^ (black bars) and control (grey bars) cardiomyocytes isolated at E12.5. Shown are the means +SEM; N = 6, *P<0.05, **P<0.01, ***P<0.001 (t test), nd, not detected.

For a more comprehensive understanding of gene expression changes in embryonic IKK^MyHC^ hearts we performed microarray analysis of embryonic ventricles at day E12.5 (S1 Table). Constitutive activation of IKK/ NF-κB resulted in a interferon type 1 signature, with a strong upregulation of interferon-regulatory factors (in particular Irf7) and the excessive expression of interferon-inducible genes like Sca1 (Ly6a/e) and Bst2. The gene expression signature of embryonic IKK^MyHC^ hearts also suggested a strong activation of Toll-like receptor (e.g. Tlr3) and Jak/ Stat signaling (e.g. Stat1, Stat2). Genes encoding chemokines (e.g. Ccl2), as well as cell adhesion molecules (e.g. Madcam1) were strongly expressed, reflecting the inflammation observed in IKK^MyHC^ hearts. Fig 7 gives an overview of the deregulated cytokines and their validation via quantitative PCR.

Several genes related to cardiac muscle architecture and function (e.g. triadin, titin, and dystrophin) were downregulated. Both apoptotic (such as Trail) and anti-apoptotic genes were upregulated in IKK^MyHC^ hearts. Transcripts of several cell cycle regulators showed a stronger expression in IKK^MyHC^ hearts, in particular the gene encoding p21, consistent with a reduction in proliferation.

### Regulators of proliferation and differentiation are disturbed in embryonic IKK^MyHC^ hearts

**Fig 8:**
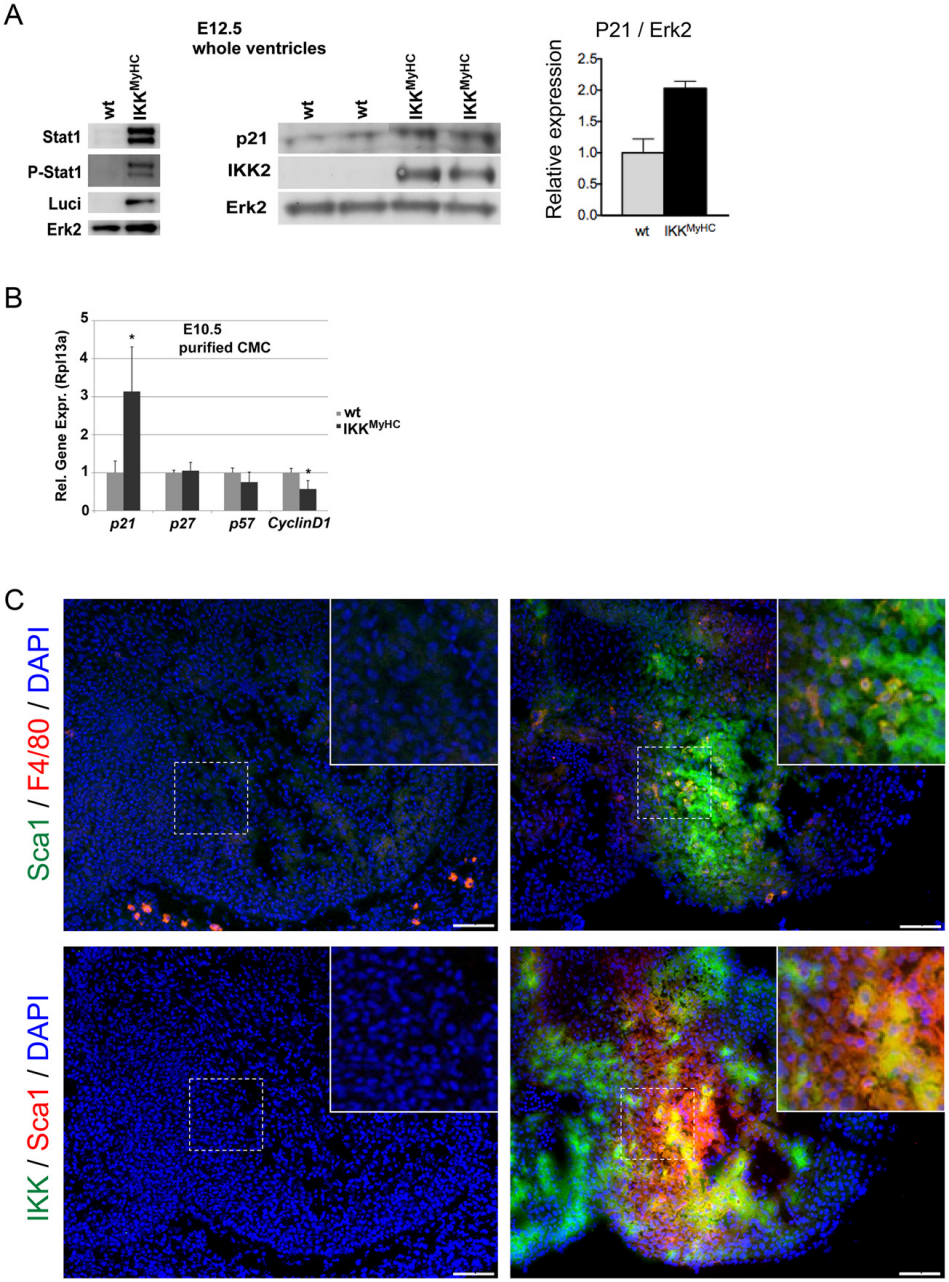
Dysregulation of regulators of proliferation and differentiation in embryonic IKK^MyHC^ hearts. (A) The expression of Stat1, phospho-Stat1, p21 and the transgenes IKK2 and luciferase was determined by Western blot, with Erk2 shown as loading control. The diagram on the right shows a quantification of the p21 immunoblot, normalized to the expression of ERK2. (B) Expression of the indicated cell cycle modulators was determined at the mRNA level using real-time quantitative PCR in purified cardiomyocytes from IKK^MyHC^ (black bars) and control (grey bars) embryos at E10.5. Shown are the means +SEM; N = 5, *P<0.05. (C) Upper panel: Staining of embryonic heart sections at E11.5 for Sca1 (green) and F4/80 (red). Lower panel: Staining of embryonic heart sections at E11.5 for the IKK2 transgene (green) and the progenitor marker Sca1 (red). DAPI was used to stain nuclei. Insets show a higher magnification of the indicated areas.

The activation of the Jak/ Stat axis suggested by the gene expression analysis was indeed confirmed on the protein level: Stat1 expression and phosphorylation was strongly enhanced in the IKK^MyHC^ embryos at E12.5 (Fig 8A). Also, the cell-cycle inhibitor p21, a target of Stat1, was upregulated at the protein level in embryonic IKK^MyHC^ hearts at E12.5 (Fig 8A).

In order to minimize the effect of infiltrating cells on these results, embryonic cardiomyocytes were isolated at E10.5, purified, and analyzed by quantitative PCR. Again, the transcript encoding p21 was upregulated (Fig 8B). In addition, the cyclin D1 transcript was downregulated.

Furthermore, we tried to elucidate the significance of the strong upregulation of the stem cell marker Sca1 (Ly6a) in the gene expression analysis. Indeed, Sca1-positive cells were a prominent feature of embryonic IKK^MyHC^, but not control hearts at day E11.5 (Fig 8C). There was only a very limited overlap between Sca1-positive cells and F4/80-positive cells, suggesting that macrophages were not primarily responsible for the observed Sca1 expression (Fig 8C, upper panel). In part, the Sca1-positive population also expressed the transgene IKK2-CA, suggesting that they constitute or contain a precursor population of cardiomyocytes with a possible function in regeneration (Fig 8C, lower panel).

### The Embryonic Lethality of IKK^MyHC^ Embryos Is Dependent on NF-κB

Finally, we wanted to address the question whether the embryonic lethality of IKK^MyHC^ embryos was in fact due to NF-κB activation or potential other pathways activated by the IKK complex. For this end, we crossed IKK^MyHC^ animals with animals expressing a mutated form of IκBα -(IκBα ^(S32A, S36A, Y42F)^), which is impervious to phosphorylation-induced degradation and thus acts as a superrepressor of NF-κB signaling[11]. Indeed, whereas only very few IKK^MyHC^ animals survived embryonic and postnatal development until weaning (constituting 1.3% of the surviving offspring instead of the expected 12.5%), IKK^MyHC^ animals expressing the IκBα -superrepressor did survive at almost normal Mendelian ratios (constituting 9.1% of the surviving offspring instead of the expected 12.5%), thus supporting the notion that IKK2 exerts its lethal effects via NF-κB signaling (Table 2).

**Table 2:** Embryonic lethality induced by constitutively active IKK2 can be prevented by an IκBα superrepressor.

MyHC-tTA	IKK2-CA	IκBα-3M	Numbers observed	Numbers expected	% observed	% expected
-	-	-	5	9.6	6.5	12.5
+	-	-	11	9.6	14.3	12.5
-	+	-	18	9.6	23.4	12.5
**Functionally wild type**			**34**	**28.9**	**44.2**	**37.5**
**+**	**+**	-	1	9.6	1.3	12.5
**IKK** ^**MyHC**^			**1**	**9.6**	**1.3**	**12.5**
**-**	**-**	+	12	9.6	15.6	12.5
+	-	+	11	9.6	14.3	12.5
-	+	+	12	9.6	15.6	12.5
**Functionally NF-**κ**B-inhibited**			**35**	**28.9**	**45.5**	**37.5**
+	+	+	7	9.6	9.1	12.5
**IKK** ^**MyHC**^ **/ I**κ**Bα-3M**			**7**	**9.6**	**9.1**	**12.5**
**Total**			**77**	**77**	**100**	**100**

The table shows the absolute number and percentage of animals born with the indicated actual and functional genotype (observed), and the corresponding expected number and percentage (expected). Only one IKK^MyHC^ animal was identified, whereas seven IKK^MyHC^ / IκB**α**-3M mice could be detected, indicating a rescue of the lethality by the IκBα-3M transgene.

## Discussion

Previously, we showed that activation of IKK/ NF-κB signaling in the adult animal led to inflammatory cardiomyopathy and heart failure [12]. Our present study now expands on these results by illuminating the consequences of IKK2 activation under the specific conditions of embryonic heart development.

Cardiac-specific expression of a constitutively active IKK2 mutant during mouse development resulted in embryonic lethality. The rather long time window during which the embryos died, ranging from E9.5 (shortly after the beginning of the activity of the α-myosin heavy chain promoter) through E16.5 with half of the embryos dead by E12.5, suggests that IKK2 activation does not interfere with a specific, chronologically strictly defined event, but rather with the general conditions required for normal heart development during a longer period of time. Alternatively, the long time window might be due to variability in the strength of transgene expression and the number of cells that express it. In any case, the rescue experiments with an IκBα superrepressor suggest that the lethality IKK2 exerts is mediated by NF-κB.

The most prominent finding in IKK^MyHC^ hearts was certainly the strong inflammatory response observed upon IKK/ NF-κB activation. The inflammatory infiltrate was characterized by F4/80-positive cells and thus monocytes/ macrophages. Gene expression analysis revealed that the signature observed in IKK^MyHC^ hearts very much resembled an interferon type I response as observed in an anti-viral response. This is consistent with the notion that IKK/ NF-κB signaling is central to the development of myocarditis, both virally induced and autoimmune [14–17]. The activation of the transcription factor STAT1, which is prominent in our model, is also known to be an early event in viral myocarditis [18].

In addition to the inflammation observed in IKK^MyHC^ hearts, the defect in compact zone formation was a prominent finding. Several biological processes could explain this defect—in particular an increase in cell death or a decrease in proliferation. Whereas cell death was indeed increased in IKK^MyHC^ embryos, this was a rather late phenomenon and therefore most likely not responsible for the disease process. In contrast, proliferation was reduced in IKK^MyHC^ embryonic hearts already at an earlier time point, suggesting that it might be responsible for the thinning of the compact zone. Indeed, proliferation is a crucial event during this time of heart development, and any disturbance might have catastrophic consequences for the developing heart [3]. This is of course much in contrast to the observations in the adult animals, where proliferation is of minor or no relevance due to the largely post-mitotic status of the adult heart [12].

We also identified a potential link between the observed inflammation and the reduction in proliferation: Among the pathways activated in this response is the JAK/ STAT pathway, which via its target gene p21 might directly inhibit proliferation [19]. The cause of the decrease in proliferation might thus indeed lie in the inflammatory environment that is so characteristic for IKK^MyHC^ hearts.

An interesting finding in IKK^MyHC^ embryonic hearts is the emergence of a Sca1-positive cell population. Recently, Sca1-positive cells have been identified as a source for myocardial renewal, albeit in the adult heart [20]. It remains to be seen whether the cells observed in IKK^MyHC^ embryonic hearts fulfill a similar and thus beneficial role, or whether they in contrast represent a lack of differentiation of cardiomyocyte progenitors, or even a dedifferentiation of cardiomyocytes. Dedifferentiation might indeed be one of the effects of IKK2, since the contractile apparatus, a hallmark of cardiomyocyte differentiation, was severely affected upon IKK/ NF-κB activation as shown by electron microscopy and immunofluorescence analyses of typical markers such as sarcomeric actinin.

Since infections are a major cause for adverse events during gestation in humans, our findings might be of relevance for a better understanding of the molecular processes in affected embryos and fetuses. According to our study, IKK/ NF-κB is a key regulator of the anti-viral response in cardiomyocytes, a finding that is very interesting in the light that viral infections (e.g. by CMV, parvovirus B19, and rubella virus) are a major problem during gestation.

## Supporting Information

S1 Table Microarray analysis reveals widespread changes in gene expression in IKK^MyHC^ embryos. The table shows the full name, the gene ID and the fold induction of genes, arranged according to their function.
(XLS)
